# Evaluating the heterogeneous effect of extended culture to blastocyst transfer on the implantation outcome via causal inference in fresh ICSI cycles

**DOI:** 10.1007/s10815-024-03023-x

**Published:** 2024-02-07

**Authors:** Yoav Kan-Tor, Naama Srebnik, Matan Gavish, Uri Shalit, Amnon Buxboim

**Affiliations:** 1 Rachel and Selim Benin School for Computer Science and Engineering, Hebrew University of Jerusalem, The Edmond J. Safra Campus Givat Ram, 9190401 Jerusalem, Israel; 2 The Center for Interdisciplinary Data Science Research, The Hebrew University of Jerusalem, The Edmond J. Safra Campus, Givat Ram, 9190401 Jerusalem, Israel; 3 Department of Cell and Developmental Biology, Hebrew University of Jerusalem, The Edmond J. Safra Campus, Givat Ram, 9190401 Jerusalem, Israel; 4 Hebrew University School of Medicine, In Vitro Fertilization Unit, Department of Obstetrics and Gynecology, Shaare Zedek Medical Center, 9103102 Jerusalem, Israel; 5 Data and Decision Sciences, Technion - Israel Institute of Technology, 3200003 Haifa, Israel; 6 Alexander Grass Center for Bioengineering, Hebrew University of Jerusalem, The Edmond J. Safra Campus, Givat Ram, 9190401 Jerusalem, Israel

**Keywords:** Assisted reproductive technologies, IVF, Embryo transfer, Causal inference, Machine learning, Digital health

## Abstract

**Purpose:**

In IVF treatments, extended culture to single blastocyst transfer is the recommended protocol over cleavage-stage transfer. However, evidence-based criteria for assessing the heterogeneous implications on implantation outcomes are lacking. The purpose of this work is to estimate the causal effect of blastocyst transfer on implantation outcome.

**Methods:**

We fit a causal forest model using a multicenter observational dataset that includes an exogenous source of variability in treatment assignment and has a strong claim for satisfying the assumptions needed for valid causal inference from observational data.

**Results:**

We quantified the probability difference in embryo implantation if transferred as a blastocyst versus cleavage stage. Blastocyst transfer increased the average implantation rate; however, we revealed a subpopulation of embryos whose implantation potential is predicted to increase via cleavage-stage transfer.

**Conclusion:**

Relative to the current policy, the proposed embryo transfer policy retrospectively improves implantation rate from 0.2 to 0.27. Our work demonstrates the efficacy of implementing causal inference in reproductive medicine and motivates its utilization in medical disciplines that are dominated by retrospective datasets.

**Supplementary Information:**

The online version contains supplementary material available at 10.1007/s10815-024-03023-x.

## Introduction

The delayed motherhood during the past two decades had led to a decline in the live birth rates from in vitro* fertilization* (IVF) treatments [1–5]. The improvement in culture conditions supports extended embryo culture with single blastocyst transfer as an alternative to cleavage-stage transfer [6], thus decreasing the medical risks that are associated with multiple pregnancies [7–9]. However, inconsistent reports indicate that the causal effect of blastocyst transfer on embryo developmental competence remains unclear [10]. In practice, extended incubation to blastocyst transfer is favorably considered only for patients with a large reserve set of embryos. For patients with a small number of embryos that are available for transfer, the risk of pre-blastulation developmental arrest leading to blastocyst transfer-cycle cancelation is higher, thus motivating cleavage-stage transfers [10]. Evidently, the assignment of the day of transfer in an embryo-specific manner to optimize implantation outcome is not directly addressed by current guidelines.

Randomized controlled trials (RCTs) suggest that blastocyst transfers can moderately improve live birth rates only of fresh cycles but not the cumulative pregnancy rate per oocyte collection cycle [11]. Despite being the method of preference for inferring causal relationships, RCTs are limited by cost, methodological and ethical considerations, thereby hindering sample size, unbiasedness toward their target embryos, and representativeness of the true distribution of embryos [12]. Alternatively, large retrospective datasets of clinically labeled video recordings of preimplantation embryo development are becoming increasingly available, owing to the utilization of time-lapse incubation systems in IVF clinics worldwide [13, 14]. Under certain special conditions, such datasets can generate compelling causal evidence that complements the evidence of RCTs [15].

Here, we present a methodological and computational framework for evaluating the heterogeneous treatment effect of extended incubation to blastocyst transfer on the implantation outcome using observational data. The dimensionality of the feature space is reduced by training a classification model for identifying the embryos that would be selected for transfer either in day 3 or day 5 transfer cycles, and propensity modeling allows for trimming the embryos that lack counterexamples. In this manner, a strong claim for satisfying the assumptions that are required for valid causal inference is provided. The conditional average treatment effect (CATE) is evaluated by training a causal forest model (CF). CF is a tree-based ensemble model that was introduced relatively recently and has gained wide adoption [16]. Using CF, the heterogeneous treatment effects are inferred by partitioning the data with respect to the multidimensional feature representation, where the splitting rule is aimed at maximizing the variance in treatment effect; this is analogous to the way decision trees’ splitting rule maximizes the outcome variance, and under the assumption of overlap CF can provably recover the treatment effect [17]. In this manner, we evaluate the so-called *transfer lift*, which quantitatively estimates the difference in the probability of the embryos to implant if transferred at the cleavage – versus – blastocyst stage. We retrospectively show that by optimizing the day of transfer of the embryos that are selected for transfer using the decision-making support tool that we report here, the implantation rate increases significantly. By providing a proof-of-concept that demonstrates the utility of causal inference in improving IVF treatments, we hope to stimulate future research and clinical validation.

## Results

### Modeling the heterogeneous treatment effect of extended incubation to blastocyst transfer on implantation outcome

**Fig. 1:**
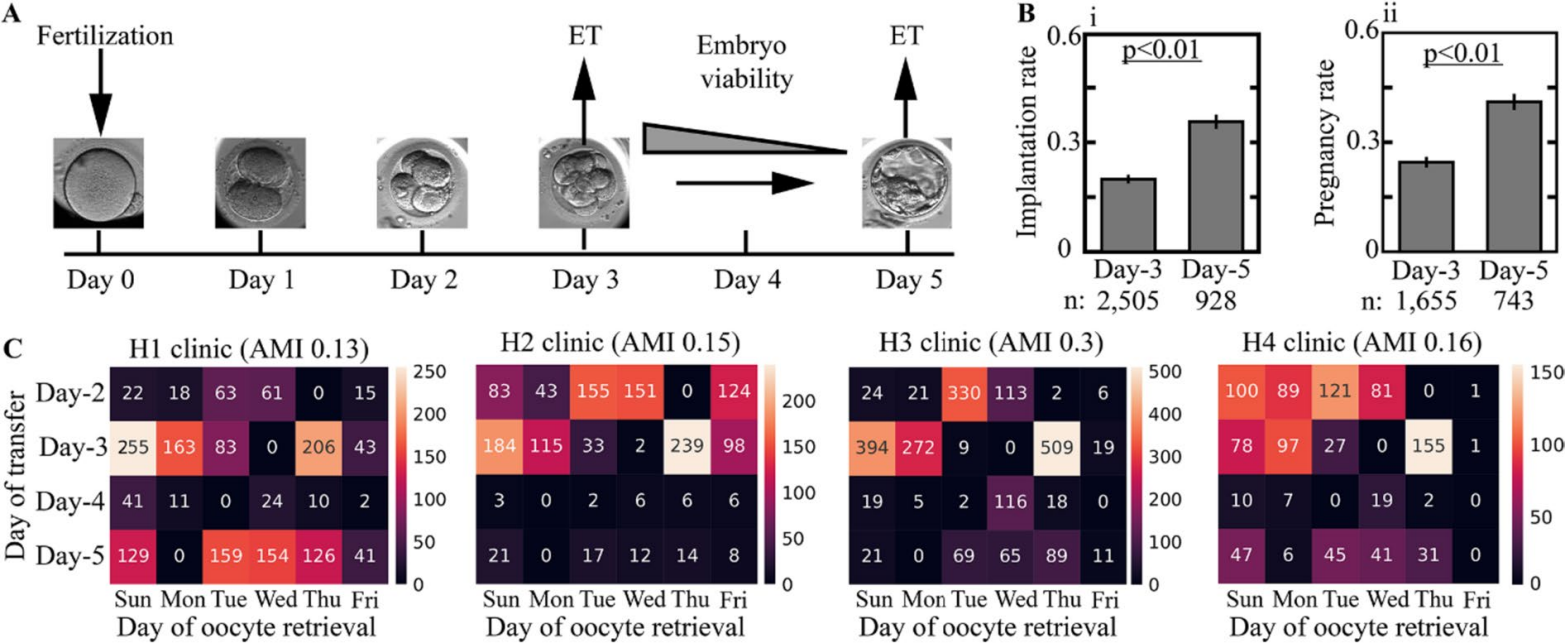
Delineating the causal model of cleavage-stage versus blastocyst transfers with respect to embryo implantation outcome in IVF-ET treatments. **A** We consider cleavage-stage transfer cycles on day 3 from fertilization and extended culture to blastocyst transfer on day 5 from fertilization. **B** Fresh day 5 transfers are associated with a higher (i) implantation rate and (ii) pregnancy rate. **C** The distributions of the day of embryo transfer from fertilization are presented versus the day of oocyte retrieval in the four data-providing medical centers. The statistical dependence between the day of embryo transfer and oocyte retrieval is quantified using adjusted mutual information (AMI)

Extended culture to blastocyst transfer in good prognosis patients is generally recommended; however, evidence-based rigid guidelines and specific criteria to direct day-of-transfer decision-making are lacking [18]. For the purpose of simplification, we consider cleavage-stage transfers that occur three days from fertilization (day 3 transfers) and blastocyst transfers that occur five days from fertilization (day 5 transfers; Fig. 1A). We report the implantation rate and pregnancy rate, which account for the fraction of implanted embryos out of all transferred embryos and the fraction of transfer cycles that resulted in one or more implanted embryos out of all transfer cycles, respectively. The implantation and pregnancy rates of day 5 transferred blastocysts were 35.7% and 41% (Fig. 1B-i,ii), thus reflecting a 70% average increase compared with day 3 cleavage-stage transferred embryos. However, these superior implantation outcome statistics cannot be taken to be causal due to potentially confounding factors in the assignment of extended incubation to blastocyst transfer, as described below [19].

Given the variation in the potential of embryos to blastulate, implant, and generate live birth, we hypothesized that the response to extended culture and to differences in endometrial synchronization that are associated with blastocyst transfers would also vary between embryos in a manner that cannot be accounted for by calculating the average treatment effect (ATE) [20–22]. In order to estimate the causal effect of extended incubation to blastocyst transfer on implantation outcome relative to day 3 embryo transfer (nontreatment assignment) from observational data, we will employ methods based on the idea of covariate adjustment (standardization) [15]. Treatment assignment is made on day 3 from fertilization with respect to the available reserve set of fertilized embryos that become available for transfer in each cycle – This is the unit of analysis. We therefore assembled a retrospective multicenter dataset of clinically labeled video files of preimplantation embryo development that we reported previously [23]. The dataset summarizes 2175 ICSI-fertilized fresh cycles that were collected from four large data-providing medical centers. It includes 3433 transferred embryos with known implantation outcomes and 18,680 embryos that had not been transferred (Table 1; “Methods”). Except for rare exceptions, our data-providing clinics operate only on weekdays (Sunday to Friday), including oocyte retrieval (OR) and ICSI that are performed on the same day. As a result, day 5 and day 3 transfers become almost excluded for patients that underwent OR on Mondays and Wednesdays, respectively (Fig. 1C). Since OR is scheduled according to follicular growth, the day of OR lacks a causal relationship with the implantation outcome [24]. This generates an exogenous source of variability with respect to the heterogeneous treatment effect for 22% of embryos in our dataset that were transferred on day 3 and 25% of the embryos that were transferred on day 5 (Fig. 1C). Finally, we note that IVF treatments are fully subsidized for the first and second children by the Israeli Ministry of Health. This policy contributes to a better representation of all socioeconomic and ethnic sectors of relevant age in our dataset.

**Table 1:** Cleavage-stage and blastocyst-stage transferred embryos

Day of transfer	Day 3 (cleavage stage)	Day 5 (blastocyst stage)
Positive implantation outcome	499	331
Negative implantation outcome	2006	597
Medical center 1	974	197
Medical center 2	630	503
Medical center 3	559	64
Medical center 4	342	164

As is well established in the causal inference literature, for covariate adjustment to yield valid causal estimates, we must examine whether the data-gathering process satisfies the *ignorability* (unconfoundedness) and *overlap* assumptions, as well as the stable unit treatment value assumption (*SUTVA*) [25]. *Ignorability* means that we have measurements of all factors that materially concurrently affect the treatment decision (day 3 vs. day 5 transfers) and the outcome (implantation); *overlap* means that every embryo could have plausibly been transferred either on day 3 or day 5; *SUTVA* means that (a) the implantation outcome of one embryo does not depend on the treatment of other embryos; and that (b) there are no multiple versions for day 3 and day 5 treatment protocols. Below, we address the degree to which the causal assumptions are satisfied, as represented in our data-gathering process.

### Ignorability assumption: delineating the confounding variables and hidden contributions

Valid causal inference from observational data requires that all the important variables that affect both treatment and outcome are represented. Ideally, this would include variables that characterize all embryos that are candidates for transfer in each cycle. One option for blocking this potential backdoor path is using a morphokinetic representation of the developmental quality of all the available embryos [26–28]. However, a typical transfer cycle includes > 5 embryos in good prognosis patients, each represented by ~ 8 morphokinetic events on day 3 from fertilization, which would require a massive dataset in order to generate counterexamples to support causal inference. Including other confounders such as maternal age would make this approach even less feasible.

Retrospective and prospective studies show that maintaining a high cumulative pregnancy rate depends on the size of the reserve set of embryos of high predicted developmental potential in addition to maternal age [29, 30]. Hence, to decrease the feature dimensionality, we tested whether the morphokinetic profiles of the entire set of available embryos can be substituted by the absolute number of low-quality and high-quality embryos in reserve set in addition to the morphokinetic profiles of the embryos that are selected for transfer per se. The feasibility of this scheme depends on the ability to identify the embryos that will eventually be selected for transfer either on day 3 or on day 5. In both cases, identifying the embryos for transfer should be made based on the embryo features that are generated by day 3. To this end, we fitted a random forest model using the morphokinetic features that were recorded by 66 h from fertilization of all the embryos that belong to day 3 transfer cycles and scored their likelihood to be selected for transfer. Indeed, the embryos that were selected for transfer in the train and test set cycles were identified with high predictive accuracy as measured by the area under the receiver operating characteristic (ROC) curves (Supplementary Fig. S1A). We executed this classifier to score the likelihood of embryos that belong to day 5 transfer cycles to be selected for transfer. Notably, classification was performed based on the morphokinetic profiles that were obtained by 66 h from fertilization of the embryos that were selected for transfer as well as the embryos that were not selected for transfer while ignoring later events. The embryos were then divided into five consecutive cohorts of equal size according to their evaluated scores; 90% of the day 5 transferred qualified the highest likelihood to be selected for transfer on day 3 had this was performed (Supplementary Fig. S1B), which probably provides an underestimation of the ability to identify the embryos for transfer due to the expected redundancy in the developmental potential between transferred and non-transferred embryos in the same cycle.

**Fig. 2:**
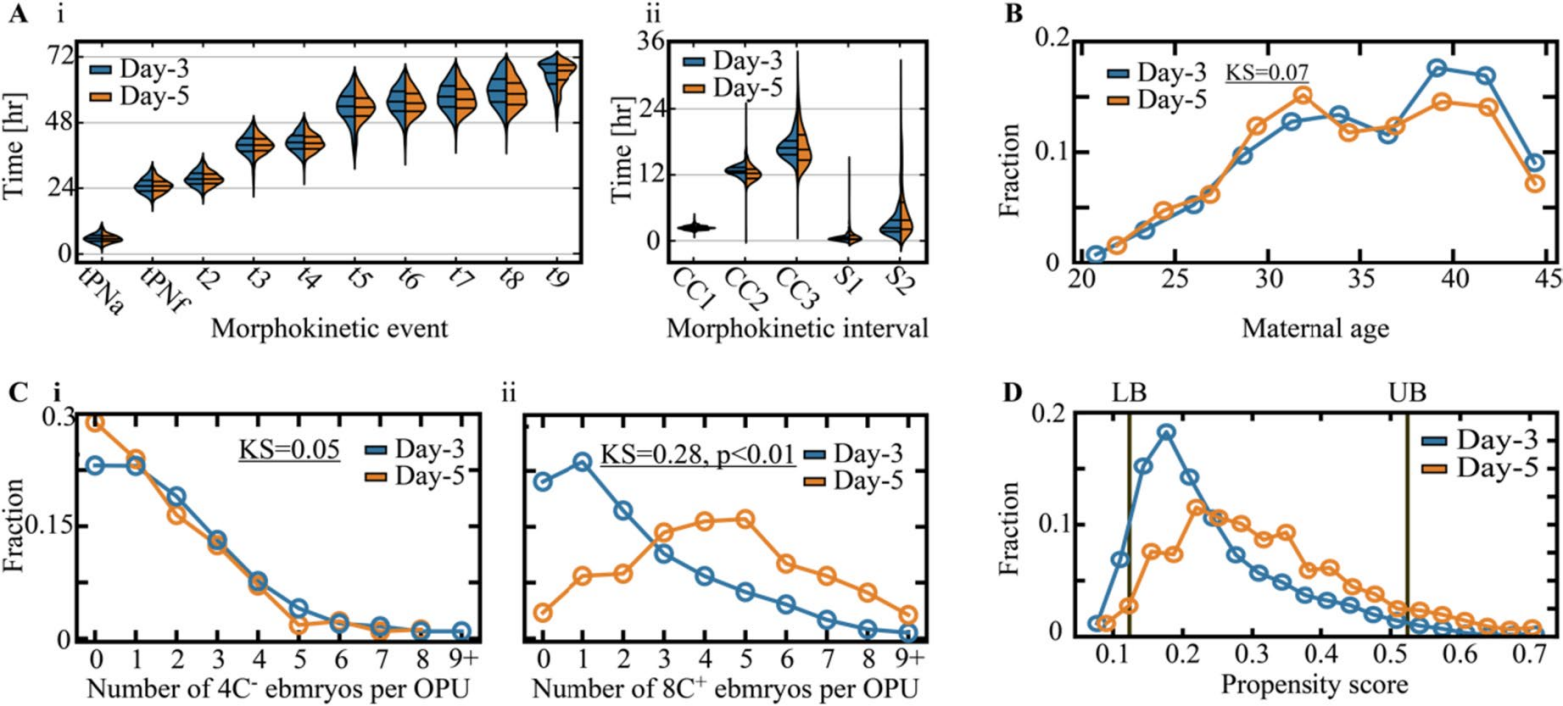
Feature analysis of cleavage-stage versus blastocyst transfers. **A** No significant differences are observed between the temporal distributions of the (i) morphokinetic events and the (ii) cell cycle and synchronization intervals of day 3 cleavage-stage transferred embryos (*n* = 1892) and day 5 transferred blastocysts (*n* = 799). KS distances < 0.1. Only high-quality embryos that reached 8-cell cleavage were included. **B** The maternal age distributions of day 3 and day 5 transferred embryos are overlapping. **C** The number of (i) low-quality (≤ 4 blastomeres) and (ii) high-quality (≥ 8 blastomeres) embryos are compared between day 3 and day 5 freshly transferred cycles. Blastocyst transfers are associated with > 3 high-quality co-cultured embryos. **D** Day 3 and day 5 propensity score distributions were derived using a logistic regression prediction model of the day of transfer. Lower bound (LB) and upper bound (UB) values for excluding non-overlapping embryos are set by the 2.5 percentile and the 97.5 percentile of the day 5 and day 3 propensity score distributions. Abbreviations: ET, embryo transfer; KS, Kolmogorov–Smirnov

Next, we characterized the effect that the specified features might have on treatment assignment. No significant differences are observed between the distributions of the morphokinetic events of day 3 and day 5 transferred embryos at 66 h from fertilization (Fig. 2A-i) and the cell cycle and synchronization intervals (Fig. 2A-ii). This indicates that embryo quality might very well be an important factor for selecting the single embryo or the few embryos for transfer but likely not for determining when to transfer – at least not as a stand-alone parameter. Parallel to embryo developmental potential, maternal age is also an important reproduction factor that is explicitly labeled for each embryo in our dataset. Maternal age is associated with an increase in chromosomal aberrations and is correlated with the size of the embryo reserve set per transfer cycle and with a decline in the developmental potential to implant in the uterus [31, 32]. Similar to the morphokinetic profiles of the embryos, there were only negligible differences in the distributions of maternal age between day 3 and day 5 transferred embryos (Fig. 2B). Hence, we conclude that maternal age does not generate a dominant effect on treatments assignment, which is independent of other factors.

Thus far, we considered the morphokinetic features of the embryos that were selected for transfer and the maternal age and found no significant differences between day 3 and day 5 transferred embryos. We next explore the effect that the predicted quality and size of the reserve set of non-transferred embryos have on the treatment assignment. Notably, the size of the reserve set was reported to correlate with the implantation outcome but not to influence the quality of the transferred embryos as assessed based on morphological scores nor to correlate with maternal age [33]. To test whether the size of the embryo reserve set affects treatment, we considered two embryo subsets: high-quality 8C^+^ embryos that consisted of ≥ 8 blastomers at 66 h from fertilization and low-quality 4C^−^ embryos that consisted of ≤ 4 blastomers at 66 h from fertilization. The size of the subsets of high-quality embryos that are valid candidates for transfer and low-quality embryos within each cycle provides an effective integrated estimation of the developmental potential of the entire cycle. No statistically significant differences are found in the number of 4C^−^ embryos (Fig. 2C-i). However, we identify a significant overrepresentation of day 5 transfer cycles that consisted of more than three 8C^+^ embryos and day 3 transfer cycles that included less than three 8C^+^ embryos (Fig. 2C-ii), which indicates that treatment assignment is favorably considered for cycles that include a sufficiently large reserve subset of high-quality embryos [34]. This policy is aimed at decreasing the risk of transfer cycle cancelation due to a developmental arrest of all the available embryos prior to blastulation. In the case of a small number of high-quality embryos on day 3, extended incubation is avoided and cleavage-stage transfers are performed. In summary, we conclude that we have measured or have proxies for most of the important and relevant confounders and proceed to discuss potential hidden confounders.

To address potential confounders that originate from medical backgrounds, we survey the most relevant clinical conditions. Repeated implantation failure (RIF) is likely underlined by non-embryonic maternal aspects, including endometrial receptivity [35]. However, the potentially confounding effect is small with a reported 10% prevalence, which is likely over-diagnosed [36]. In the case of past preterm labor, a single embryo transfer policy is favorable; however, there are no specific guidelines for choosing cleavage-stage or blastocyst transfer [37]. Similarly, single embryo transfer is recommended in patients with Mullerian anomalies (e.g., unicornuate uterus), which are rare congenital conditions that are characterized by an increased risk of miscarriage and preterm delivery, whereas no effects are known on the implantation potential in IVF treatments [38]. Hence, the impact of these main medical background conditions on day-of-transfer treatments is either insignificant or nonspecific. We therefore believe that all important factors affecting both treatment and outcome are represented in our dataset, leading us to conclude that the *ignorability* assumption is very nearly satisfied by our data-gathering process.

### Overlap and SUTVA assumption: limitations to inferring the heterogeneous treatment effect on implantation outcome

To explore the extent of *overlap* between cleavage-stage and blastocyst transfers, we fit a logistic regression model for predicting the treatment assignment using the parameters described above: (1) the morphokinetic events from the time of pronuclei appearance (tPNa) to nine blastomere cleavage, (2) maternal age, (3) the number of 4C^−^ embryos, and (4) 8C^+^ embryos at 66 h from fertilization [39]. With respect to these feature vectors, cleavage-stage and blastocyst transfers were only partially separated. This is indicated by the propensity score, which quantifies the day-of-transfer prediction probability (Fig. 2D). We find that excluding (trimming) the embryos with propensity below the 2.5 percentile of day 5 transferred embryos or above 97.5 percentile of day 3 transferred embryos removes all cases that lack counterexamples, thus satisfying the *overlap* assumption between conditions among the remaining embryos [40]. To characterize these embryos that are excluded from causal analysis, we compared them with the remaining embryos. There were no differences in the morphokinetic events (Supplementary Fig. S2A-i,ii), maternal age distributions (Supplementary Fig. S2B-i), and the number of 4C^−^ embryos (Supplementary Fig. S2B-ii) between excluded and remaining embryos. However, we found that the excluded embryos were characterized either by a relatively small number (≤ 1) or a very high number (≥ 9) of 8C^+^ embryos (Supplementary Fig. S2B-iii).

Next, we consider the degree to which the SUTVA assumption is satisfied. Clearly, the implantation outcome of embryo transfer in one patient is not affected by the treatment assignment in other patients. Secondly, the four data-providing medical centers adhere to the same European Society of Human Reproduction and Embryology (ESHRE) guidelines, which decreases the potential confounding contributions of variation. To further minimize potential sources of variation between treatments, we included only ICSI-fertilized fresh transfer cycles of embryos that were cultured in the same automated time-lapse incubator (EmbryoScope time-lapse incubator version D, Vitrolife A/S, Denmark) under the same controlled environmental conditions.

We, therefore, conclude that similar to the ignorability and overlap assumptions, both *SUTVA* requirements are satisfied by our data-gathering process, which provides strong evidence that the dataset supports the possibility of valid causal inference for evaluating the causal effect of extended incubation to blastocyst transfer on implantation outcome. Our ability to infer these causal effects is further supported by the exogenous source of variability presented above [15, 41].

### Fitting a causal forest model for evaluating the heterogeneous day-of-transfer treatment effect of extended culture to blastocyst transfer on embryo implantation

Establishing a strong claim for satisfying the assumptions needed for valid causal inference from observational data qualified the fitting of a CF model to evaluate the heterogeneous day-of-transfer treatment effect using the maternal, cycle, and embryo features and the overlapping dataset as validated by the propensity model [16]. Using CF, we calculated the so-called *transfer lift* of the embryos at 66 h from fertilization. The *transfer lift* is the conditional average treatment effect (CATE); namely, it is the estimated implantation potential (in terms of probability, between 0 and 1) of individual embryos if transferred on day 5 minus the same potential if transferred on day 3. The *Transfer lift* ranges between − 1 and 1, where positive *transfer lift* indicates that the implantation potential is predicted to be higher if transferred on day 5 and vice versa. A mathematical description of CF fitting and evaluating the *transfer lift* is provided in the “Materials and Methods” section.

**Fig. 3:**
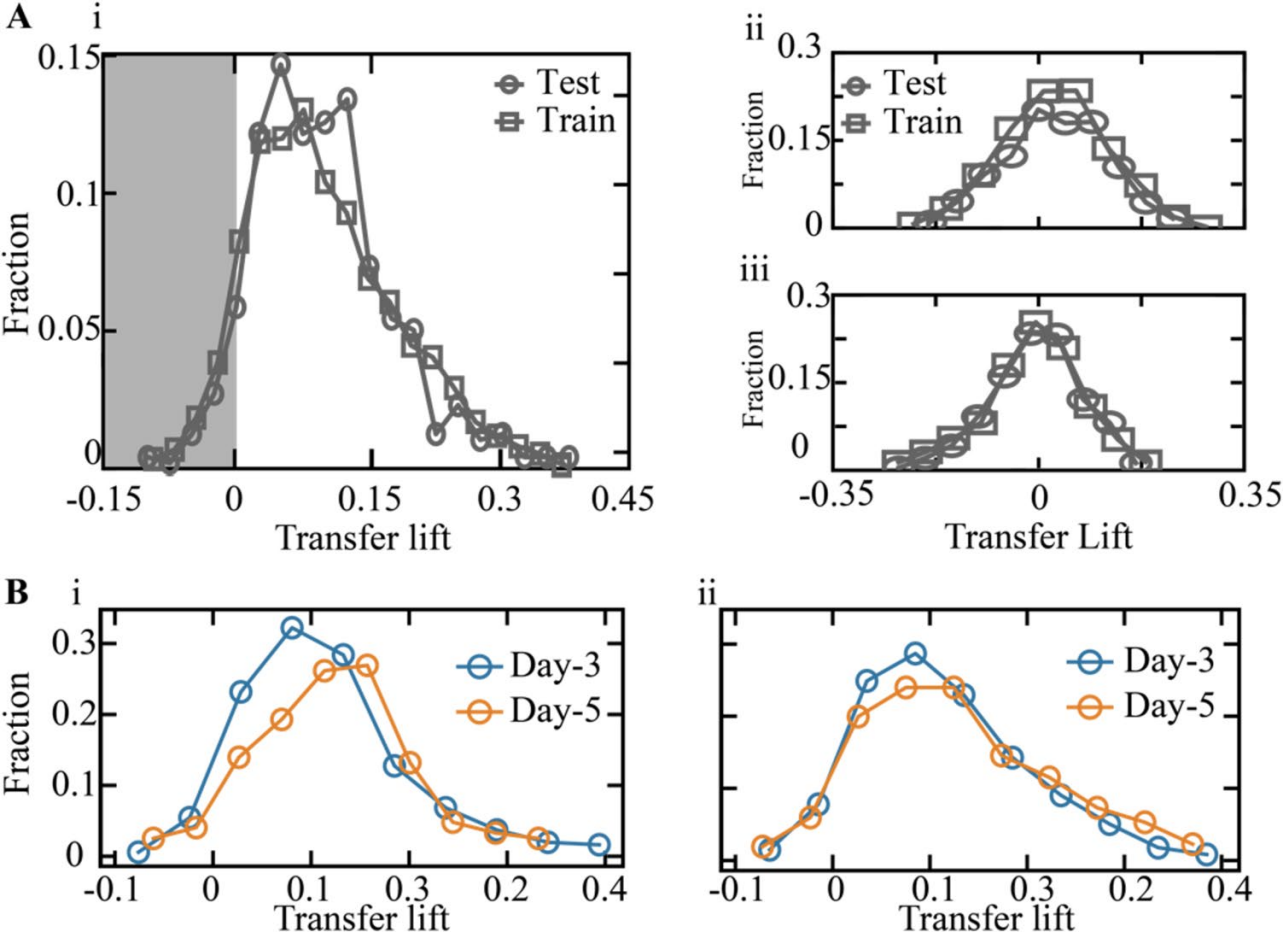
The *transfer lift* measures the difference in the implantation potential of embryos if transferred at the blastocyst stage relative to the cleavage stage. **A** (i) The obtained test-set and train-set *transfer lift* distributions are asymmetric about zero, consisting of embryos with negative (gray background) and with positive (white background) *transfer lift* values. As a control, *transfer lift* was evaluated after randomly permuting (ii) the implantation outcome and (iii) the day-of-transfer labels. The differences of the implantation outcome (ii) and the day-of-transfer (iii) permuted distributions from the non-permuted *transfer lift* distribution (i), as evaluated using KS statistics, was 0.34 and 0.51 KS distance respectively, and *p*-value < 0.01. **B** The *transfer lift* distributions of nontreated and treated embryos overlap, as quantified by the Kolmogorov–Smirnov distance for (i) the test set (KS = 0.12) and (ii) the train set (KS = 0.12). STD: standard deviation

The *transfer lift* distributes between − 0.1 and 0.35 with an average value $\pm$ standard deviation of $0.1\pm 0.07$ (test set) and $0.1\pm 0.08$ (train set; Fig. 3A-i). While the majority of embryos were scored a positive *transfer lift*, a negative *transfer lift* was evaluated for 6.3% ($N=30$) of the test-set embryos and 8.5% ($N=49$) of the train-set embryos with up to 0.1 higher estimated probability for implantation if transferred on day 3. As a control, we fitted a CF model after randomly permuting the implantation outcome (Fig. 3A-ii) and the day of transfer (Fig. 3A-iii). In both cases, the *transfer lift* is distributed symmetrically about zero. As expected, we obtained symmetric distributions with equal representation of positive and negative *transfer lift* embryos that were statistically significantly separated from the non-permuted distributions. To verify generality and test the potentially confounding effects that might be generated by the differences between clinics and embryo transfer protocols, we compared the *transfer lift* distributions of the embryos from the four data-providing medical centers (Supplementary Fig. S3A-i,ii) and also compared the *transfer lift* distributions of single-embryo-transferred embryos and double-embryo-transferred embryos (Supplementary Fig. S3B-i,ii). Finally, the *transfer lift* distributions of treated and nontreated embryos also overlapped, thus verifying the lack of bias in the day-of-transfer treatment assignment by current IVF policies (Fig. 3B-i,ii).

**Fig. 4:**
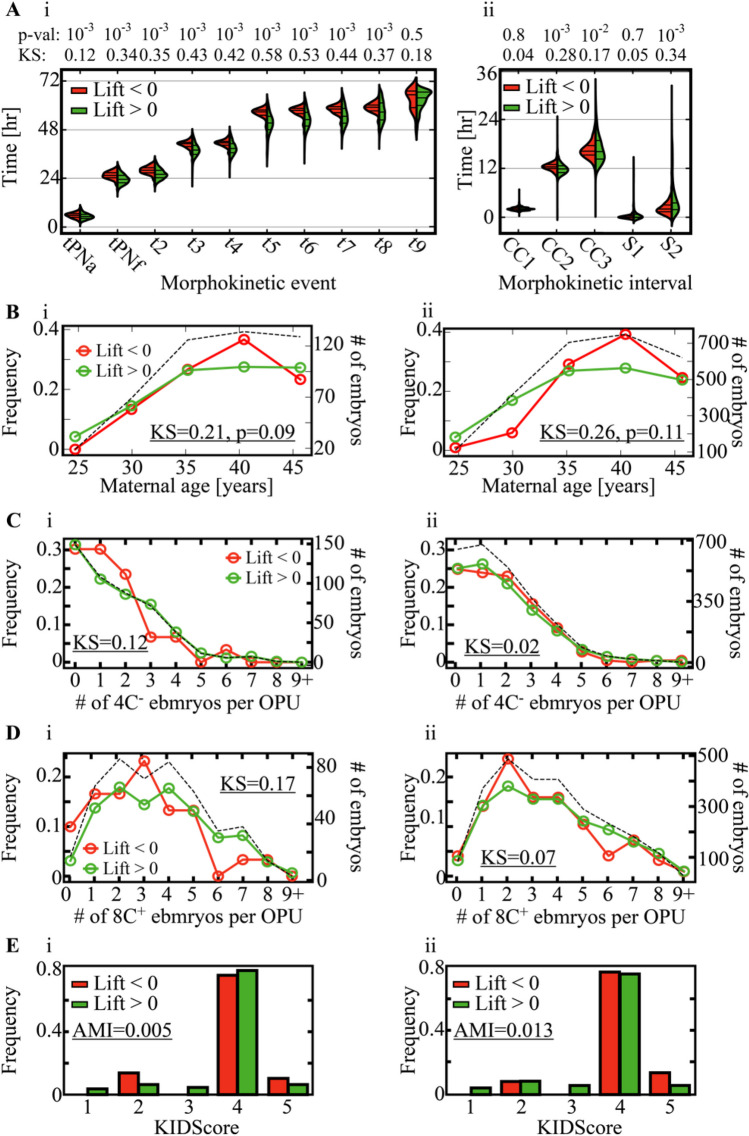
Feature analysis of positive versus negative *Transfer Lift* embryos. (**A**) A comparison of the temporal distributions of the (i) morphokinetic events and the (ii) cell cycles (CC1-to-CC3) and synchronization (S1, S2) intervals at 66 h from fertilization indicates that negative *transfer lift* embryos develop slower than positive *transfer lift* embryos. (**B**) Comparison of the maternal age distributions of (i) test-set and (ii) train-set embryos. (**C**) Comparison of the number of low-quality embryos (4C^−^ at 66 h from fertilization) per oocyte retrieval of positive and negative *transfer lift* (i) test-set and (ii) train-set embryos. (**D**) Comparison of the number of high-quality embryos (8C^+^ at 66 h from fertilization) per oocyte retrieval of positive and negative *transfer lift* (i) test-set and (ii) train-set embryos. (**E**) Comparison of day 3 KIDScore ranking of (i) test-set and (ii) train-set embryos. The dependence between the *transfer lift* distributions and the KIDScore distributions is quantified via AMI. The total number of embryos is depicted by the dashed lines in (**B**, **C**, and **D**). Abbreviations: p-val, p-values; KS: Kolmogorov–Smirnov distances; AMI: adjusted mutual information

Next, we characterize the differences in the developmental properties of negative and positive *transfer lift* embryos. To this end, we compared the distributions of the morphokinetic events of 8C^+^ embryos in each group, which reveals that the negative *transfer lift* embryos are characterized by slower developmental dynamics mostly in the time of five-cell and six-cell cleavage events (*t5* and *t6*) relative to positive *transfer lift* embryos (Fig. 4A-i,ii). No significant differences between positive and negative *transfer lift* embryos were observed in maternal age (Fig. 4B-i,ii), the number of low-quality 4C^−^ embryos (Fig. 4C-i,ii), and high-quality 8C^+^ embryos (Fig. 4D-i,ii), as evaluated based on Kolmogorov–Smirnov statistics. Finally, we compared the assessment of embryo developmental potential using the broadly used day 3 KIDScore classification tool (Fig. 4E-i,ii) [42]. Despite the abovementioned morphokinetic gaps between positive and negative *transfer lift* embryos, the KIDScore distributions overlapped, and no significant statistical dependence was obtained between the *transfer lift* and the KIDScore distributions as verified using adjusted mutual information analysis. In summary, the statistical indications that we generated here suggest that the *transfer lift* is a property of individual embryos that is independent of maternal age, oocyte retrieval statistics, and predicted embryo quality.

**Fig. 5:**
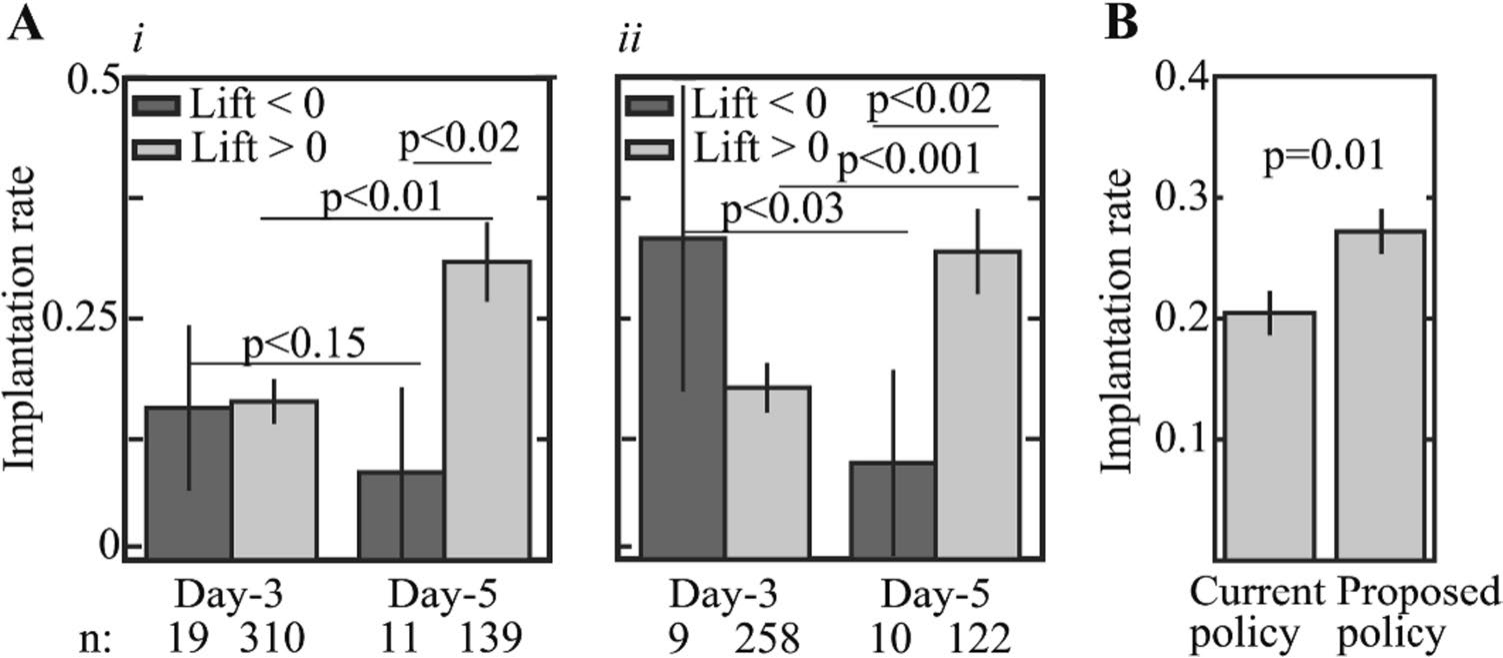
The *transfer lift* measures the heterogeneous day-of-transfer treatment effect. **A** The implantation rates of negative and positive *transfer lift* embryos that were transferred on day 3 and day 5 are compared. Average implantation rates are presented (i) for all test-set embryos and (ii) across high-quality embryos only (8C + at 66 h). Error bars represent STD. *P*-values were evaluated using permutation testing (1000-fold randomly sampled permutations). **B** Retrospective comparison of the actual implantation rate (current proposed) and the estimated implantation rate by the proposed policy of transferring negative and positive *transfer lift* embryos at day 3 and day 5. Error bars depict STD. KS: Kolmogorov–Smirnov. STD: standard deviation

To assess the estimated treatment effect on the actual treatment outcome, we compared the implantation rates of negative and positive *transfer lift* test-set embryos. The average implantation rate of positive *transfer lift* embryos was higher when transferred on day 5, and the average implantation rate of negative *transfer lift* embryos was higher when transferred on day 3 (Fig. 5A-i). The estimated treatment effect on the implantation outcome of high-quality 8C^+^ embryos was larger and statistically more significant (Fig. 5A-ii). To account for the limitations that may be associated with the size of the available test set, we quantified the statistical significance by performing 1000-fold randomly sampled permutation testing and further validated it using a Wilcoxon rank-sum test (Methods). Finally, we addressed the sensitivity to the question of whether the effect of the clinical site is random or not. To this end, we fit a second logistic regression model to calculate the propensity score distributions of cleavage-stage versus blastocyst transfers in which the medical center was included as a dummy variable (Supplementary Fig. S4A). As before, non-overlapping embryos with propensity scores below the 2.5 percentile of the day 5 distribution and above the 97.5 percentile of the day 3 distribution were excluded. A CF model was then fitted using the same feature vectors including the medical center dummy variable to calculate the *transfer lift* at 66 h from fertilization as described above. Satisfyingly, the differences in the implantation outcome between negative and positive *transfer lift* embryos that were transferred on day 3 versus day 5 were reproduced across all test-set embryos (Supplementary Fig. S4B-i) and 8C^+^ embryos (Supplementary Fig. S4B-ii). We therefore conclude that the effect of the clinical site is not significant.

The evaluation of the heterogeneous treatment effect indicates that the implantation rate could be increased by transferring negative *transfer lift* embryos on day 3 and positive *transfer lift* embryos on day 5. To test this proposed policy, we retrospectively re-adjusted the implantation outcome of positive *transfer lift* embryos that were transferred on day 3 and negative *transfer lift* embryos that were transferred on day 5 by adding the absolute *transfer lift* values. This re-adjustment scheme is based on the fact that the *transfer lift* measures the difference in the probability of the embryos to implant if transferred at the cleavage stage and the blastocyst stage. In comparison with the current policy, the proposed policy is retrospectively estimated to have increased the average implantation rate from 0.2 to 0.27, which is a 32% increase relative to the current policy (Fig. 5B).

## Discussion

Incorporating evidence-based medicine in IVF-embryo transfer treatments is hindered by ethical constraints on clinical trials and bias in observational studies [43]. To overcome these limitations, we fit a causal forest model and evaluate the *transfer lift*, which measures the heterogeneous treatment effect of extended culture to blastocyst transfer on the implantation outcome. Using an expansive retrospective multicenter dataset, we confirm that extended culture to blastocyst transfer is considered in the case that the size of the reserve set of high-quality cleavage-stage embryos is high on day 3. In this manner, the risk of transfer cycle cancelation due to the lack of embryos that properly develop into functional blastocysts by day 5 is decreased. However, the response of the embryos that are selected for transfer to extended culture for blastocyst transfer is the determinant factor. Hence, we considered the potential confounders and hidden confounders, identified an exogenous source of variability, and demonstrated the likelihood that the required causal assumptions are satisfied by the data-generating process and can thus support causal inference analysis to calculate the heterogeneous day-of-transfer treatment effect on implantation potential [15, 44, 45].

The main confounding variables that affect treatment and outcome should be included for evaluating the propensity score and fitting the causal forest model. The degree to which the confounding factors are included and the limitations that are imposed by not including all potential variables is inherent to causal inference. The features that we include can be divided into three categories: (1) maternal age, which is a recognized factor that is associated with a decline in reproductive potential [46]. (2) The developmental potential of the embryos that are selected for transfer is marked by their morphokinetic events at 66 h from fertilization. (3) The embryo reserve set is characterized by the number of high (8C^+^) and low (4C^−^) quality embryos that are available for transfer in each cycle. Notably, we show that the embryos that are to be selected for transfer on day 5 can be predicted based on their day 3 morphokinetic profiles, which is a preliminary step that allows for defining the unit of analysis, setting the number of low and high quality non-transferred reserve-set embryos, and evaluating the *transfer lift*.

Unfortunately, IVF history, medical background, and other potentially confounding variables are not documented in our dataset. Nevertheless, the treatment assignment of 22 to 25% of the transferred embryos obeys the exogenous source of variability and is, therefore, independent of potential hidden confounders. The multicentral distribution of our dataset contributes to its generality. However, this is compromised by the fact that just over half of the treated embryos belong to one out of the four data-providing medical centers. Other limitations to the conclusions of our model are imposed by the exclusion of the transfer cycles that lacked counterexamples and obtained relatively high (day 3 transfers) or low (day 5 transfers) propensity scores. This propensity rule cannot be interpreted directly in terms of medical and developmental parameters. However, the propensity scores of future transfer cycles can be easily determined using our regression model to verify whether it is included or excluded from our causal model. Finally, transfer cycles that had been selected for extended culture to day 5 but were eventually canceled due to the failure of generating blastocysts are not included in our database. This generates an information bias in our data collection scheme, which is a limitation that is shared by causal models across different disciplines [47]. Owing to the underrepresentation of day 5 transfer cycle cancelations, the average implantation rate of day 5 transfers is likely overestimated, and the evaluation of ATE presents an upper bound. It may also cause an overestimation of the improvement in the implantation rate using the proposed policy relative to the current policy. However, the evaluation of the fraction of embryos with negative *transfer lift* is likely underestimated since canceled transfer cycles might have generated a positive outcome only if the embryos had been transferred on day 3. This would contribute to the increase in the implantation rate by using the proposed policy compared with the current policy. Finally, our work relies on observational data; despite our best efforts and the uniqueness of our dataset, we can never rule out the possibility of some unmeasured confounder inducing spurious correlations and misleading our model.

The molecular mechanisms that underlie the *transfer lift* cannot be determined via a computational vision-based approach and are thus beyond the scope of this work. However, differences in the *transfer lift* suggest that embryos respond heterogeneously to the environmental conditions that are set by the culture medium and/or in the uterus. Specifically, negative *transfer lift* embryos are characterized by slower morphokinetics relative to positive *transfer lift*, which might introduce differences in embryo-endometrium synchrony. Compared with positive *transfer lift* embryos, negative *transfer lift* embryos are likely more sensitive to the extended culture environment during morula compaction and blastulation. On the other hand, positive *transfer lift* embryos might gain from a better synchronization with the uterine environment if transferred as blastocysts during the window of implantation [20].

In summary, we present a decision support tool for estimating patient-specific day-of-transfer treatment effects in a manner that is complementary to existing classifiers of the potential of embryo implantation [23] and 1st-trimester miscarriage [48]. We estimate that optimizing the day of transfer of the embryos that are selected for transfer by the proposed policy is expected to significantly improve the implantation rate, thus supporting single embryo transfers while shortening the time to pregnancy. In this context, our causal analysis provides a proof of concept and motivates future research. However, medical utilization requires overcoming the limitations that are imposed by our data collection scheme, as discussed above using larger and unbiased datasets. We hope our work will spur clinical experiments based on per-embryo treatment protocols.

## Materials and methods

### Dataset

In this work, we used a multicenter dataset consisting of clinically labeled embryos that were included in ICSI-fertilized fresh IVF cycles, as previously reported. Ethical approval was obtained by the Investigation Review Boards of the data-providing medical centers: Hadassah Hebrew University Medical Center IRB number HMO 558–14; Kaplan Medical Center IRB 0040–16-KMC; Soroka Medical Center IRB 0328–17-SOR; and Rabin Medical Center IRB 0767–15-RMC [23]. In total, embryos from 2175 fresh cycles were included, consisting of 3433 transferred embryos with known implantation outcomes (Table 1), which does not include DETs with an indication of only one implanted embryo. Successful implantation was determined based on the measured number of gestational sacks on week five of pregnancy. In addition, the dataset included 18,680 embryos from the same cycles that had not been transferred (12,091 embryos from day 3 transfer cycles and 6589 from day 5 transfer cycles). The embryos were cultured in nine time-lapse incubators (TLIs) located in the four medical centers specified above. Morphokinetic annotation of the transferred embryos was performed and validated as reported by trained embryologists adhering to established protocols [23]. The assignment of 8C^+^ and 4C^−^ non-transferred embryos was performed using automated annotation [49]. The distributions of the TLIs, multiple transfers, maternal age, and implantation outcome (parsed by medical center) are provided by Kan-Tor et al. [23].

### Random forest

A random forest model for predicting the likelihood of embryos to be selected for transfer on day 3 from fertilization was implemented using the scikit-learn python package. Model fitting was performed using the embryos from all day 3 transfer cycles, which were labeled as transferred or non-transferred, and the morphokinetic profiles of their events that occurred up to 66 h from fertilization were used as features. Missing morphokinetic annotations were assigned a zero value. The dataset was randomly divided into train (92%) and test (8%) sets. Meta parameter search was performed using a grid search. The selected parameters were class weight balanced, split criterion entropy, max tree depth 7, max number of features using the log2 rule, and the number of estimators was 80. Other parameters were set to default.

### Logistic regression

The propensity score models for evaluating the day of transfer were fitted as described in the “Results” section using a logistic regression model that was implemented using the scikit-learn python package with L2 penalty. Day 3 and day 5 transferred embryos had been randomly divided into train (85%) and test (15%) sets. Fitting was performed using the following features: (1) the morphokinetic profiles from time of pronuclei appearance (tPNa) to nine blastomere cleavage (t9) as illustrated in Fig. 2A, (2) maternal age, (3) the number of 4C^−^ embryos, and (4) 8C^+^ embryos at 66 h from fertilization. Missing morphokinetic annotations were assigned the average values of each event.

### Causal forest

The CF model was trained using the Generalized Random Forests package [16], which is implemented in the R programming language [50]. The model parameters were selected based on a fivefold cross-validation scheme. CF training was performed with 300 trees and a minimum of 20 embryos per leaf. A mathematical formulation of the model is provided below.

For a given embryo $$i$$, the feature vector $${X}_{i}$$ consists of the morphokientic profile, maternal age, and the number of high- and low-quality co-cultured embryos at 66 h from fertilization as defined in the main text (Supplementary Fig. S5A). Missing morphokinetic annotations were assigned a zero value. The treatment indicator divides the embryos into “non-treated” day 3 transferred embryos ($${W}_{i}=0$$) and “treated” day 5 transferred embryos ($${W}_{i}=1$$). The individual treatment response $${Y}_{i}$$ corresponds to negative implantation ($${Y}_{i}=0)$$ or positive implantation ($${Y}_{i}=1)$$ outcome. Using feature vectors $${X}_{i}$$, we trained a CF model consisting of $$B$$ causal decision trees (CDTs; Supplementary Fig. S5B) [16]. Co-representation of day 3 and day 5 transferred embryos was verified along the entire feature vector range by excluding the embryos that lacked counterexamples with overlapping propensity scores (Fig. 2D). Heuristically, each CDT divides the embryos according to $${X}_{i}$$ with respect to $${W}_{i}$$ into discrete leaves (Supplementary Fig. S5C). Within a given leaf $${L}_{j}^{b}$$, the treatment effect $${\widehat{\tau }}_{b}\left(x\right)$$, $$x\in {L}_{j}^{b}$$, is defined by the average response of the treated embryos minus the average response of the non-treated embryos:

$${\widehat{\tau }}_{b}\left(x\right)=\frac{1}{\left|{E}_{j}^{b,1}\right|}\sum_{i\in {E}_{j}^{b,1}}{Y}_{i}-\frac{1}{\left|{E}_{j}^{b,0}\right|}\sum_{i\in {E}_{j}^{b,0}}{Y}_{i}$$

where ${E}_{j}^{b,k}=\left\{i|{w}_{i}=k, {X}_{i}\in {L}_{j}^{b}\right\}$. The conditional average treatment effect of any embryo with features $x$ is evaluated by the *transfer lift*, which is the averaged treatment effect across all $B$ causal trees in the forest:

$$\widehat{\tau }\left(x\right)=\frac{1}{B}\sum_{b\in B}{\widehat{\tau }}_{b}\left(x\right)$$

The *transfer lift* measures the difference in the implantation potential in case it was transferred on day 5 relative to day 3. The *transfer lift* ranges between − 1 and 1, where positive values account for embryos whose implantation potential is higher if transferred on day 5 and vice versa.

### Statistical analysis

Graphs and heatmaps were plotted using matplotlib and seaborn python packages. Student’s *t*-test, Wilcoxon rank-sum test, and Kolmogorov–Smirnov statistics were calculated using the Python SciPy package. Adjusted mutual information was calculated using the scikit-learn python package with the arithmetic average method. The statistical significance of the differences in implantation rates between day 3 and day 5 transferred embryos with negative and positive *transfer lift* (Fig. 5A) was determined using a permutation test, with the null hypothesis that the observed low implantation rate in the smaller group is due to chance. In each test, 1000 permutations were randomly sampled. Our results are validated using a Wilcoxon rank-sum test that generated comparable results.

### Supplementary Information

Below is the link to the electronic supplementary material.

Supplementary file1 (DOCX 736 KB)

## Data Availability

The copyrights of the code are owned by Yissum – the technology transfer company of The Hebrew University of Jerusalem. Requests can be sent to A.B. The clinical data are owned by the Hadassah Medical Center and the Clalit Health Services. Restrictions apply to the availability of these data, which were used anonymously under ethical agreements with each clinic separately for this study and so are not made publically available.
