# Supplementary material for: Evaluating the heterogeneous effect of extended culture to blastocyst transfer on the implantation outcome via causal inference in fresh ICSI cycles
Source: J Assist Reprod Genet. 2024 Feb 7;41(3):703–15. doi: 10.1007/s10815-024-03023-x (PMC10957840; doi:10.1007/s10815-024-03023-x)
Supplement: Supplementary file 1 — Supplementary file1 (DOCX 736 KB) [file 10815_2024_3023_MOESM1_ESM.docx]

**Supplementary information**

| 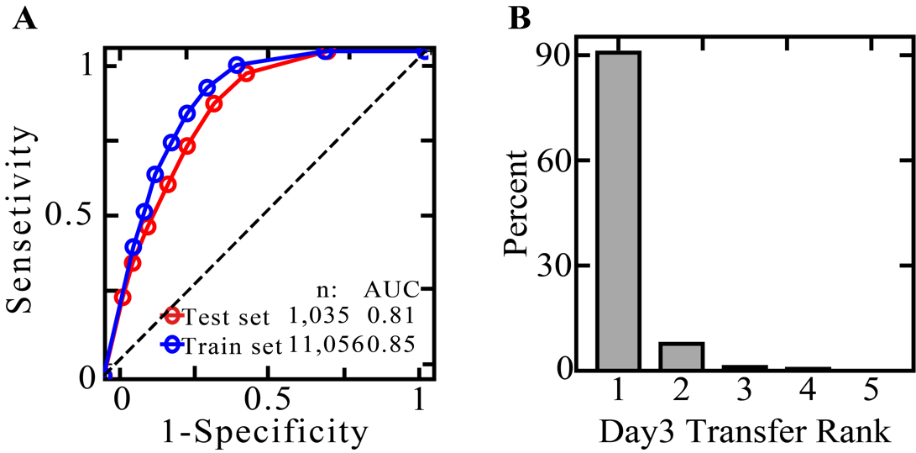 |
| --- |
| **Figure S1 \| Day-3 and Day-5 transfer candidates are overlapping:** **A,** A random forest model was trained to predict the likelihood of embryos to be selected for transfer on Day-3 from fertilization. ROC curves are provided with AUC for train and test sets. **B,** The distribution of the inferred likelihood of Day-5 transferred blastocysts to be selected for Day-3 transfer had Day-3 transfers been performed is presented. Rank-1: highest likelihood. Rank-5: lowest likelihood. |

| 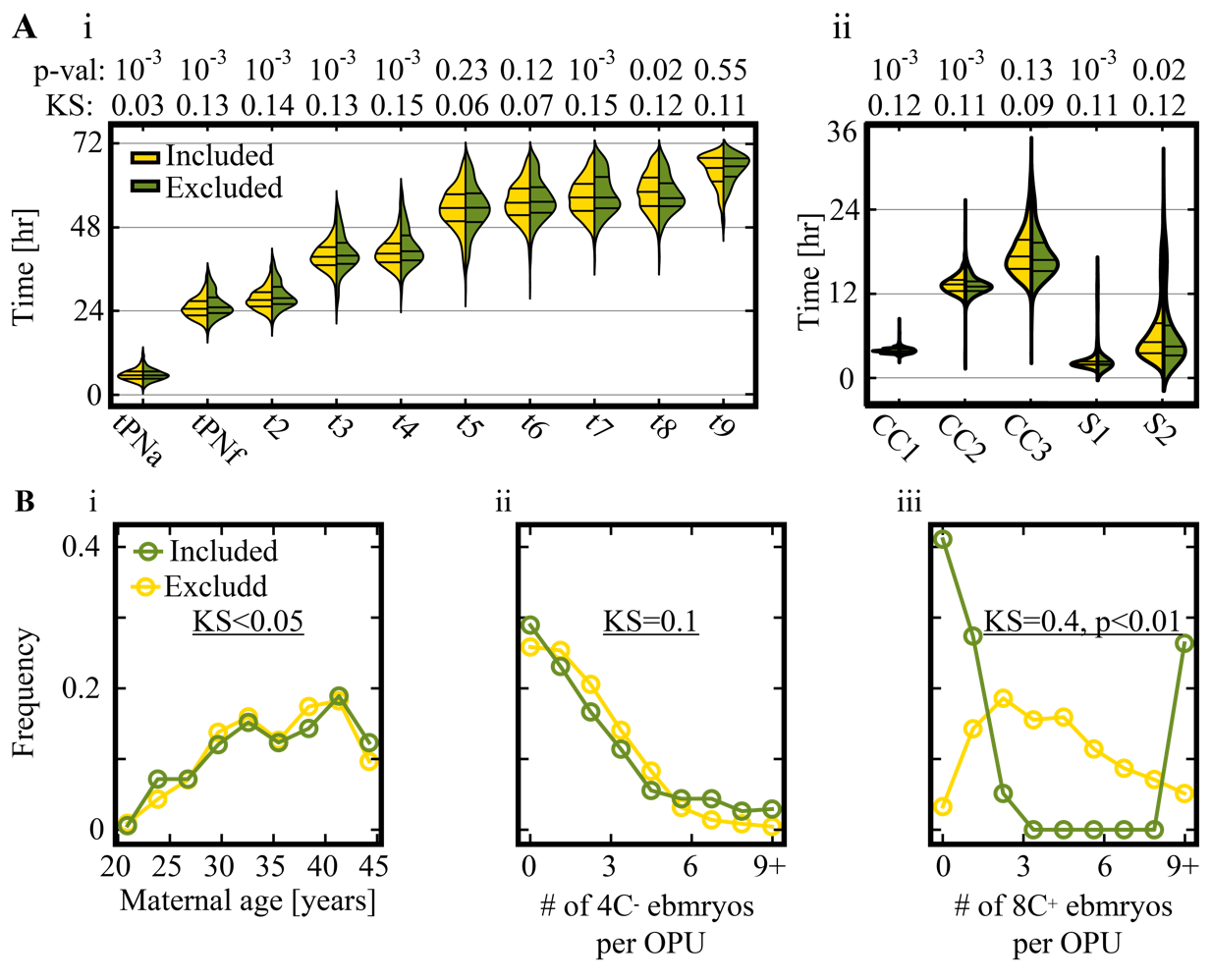 |
| --- |
| **Figure S2 \| Characterization of the embryos excluded from causal analysis.** The embryos with propensity scores outside the counterexample range (excluded) are characterized via comparison of the features used for causal inference with the remaining embryos (included). **A,** A comparison between the temporal distributions of the (i) morphokinetic events, and the (ii) cell cycle (CC1-to-CC3) and synchronization (S1, S2) intervals as evaluated 66 hours from fertilization. **(B),** Comparison of the (i) maternal age distributions, (ii) number of low quality embryos (4C^-^ at 66 hours from fertilization), and (iii) number of high quality embryos (8C^+^ at 66 hours from fertilization) per cycle. Included embryos: n=2,254 (Day-3) and n=829 (Day-5). Excluded embryos: n=251 (Day-3) and n=99 (Day-5). Kolmogorov-Smirnov distances (KS) are specified. |

| 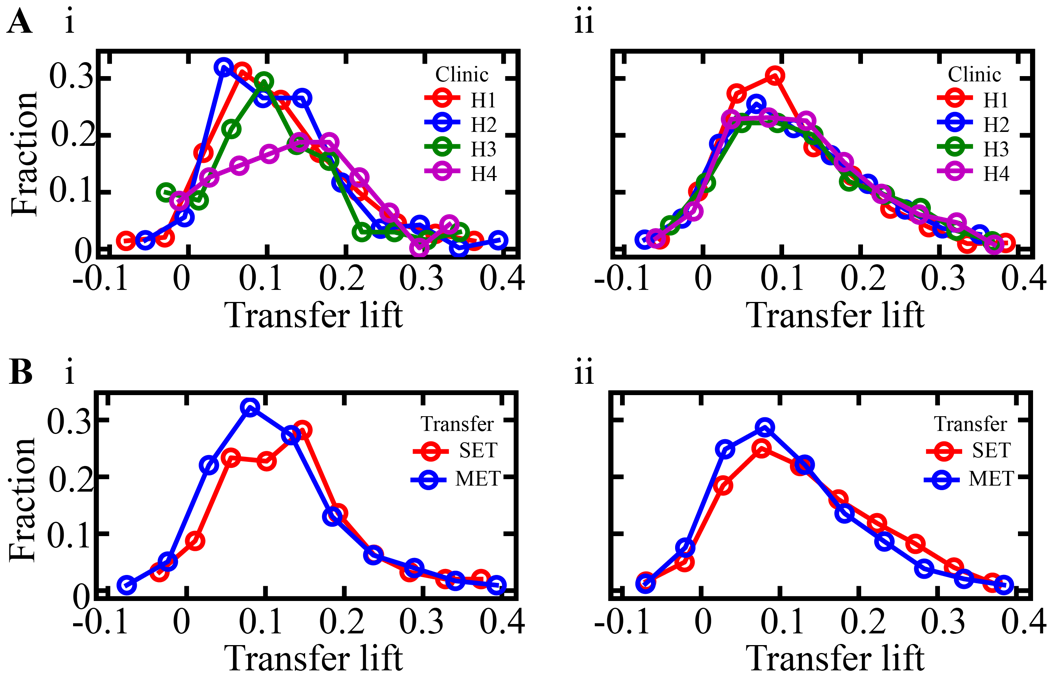 |
| --- |
| **Figure S3 \| The *Transfer Lift* is stable across medical and clinical parameters: A** Comparison of the *Transfer Lift* distributions of the embryos that were obtained from H1-through-H4 IVF clinics. Peak-to-peak KS distance across clinics of (i) test-set and (ii) train set embryos was 0.24 and 0.12, respectively. **B,** Comparison of the *Transfer Lift* distributions of single-embryo transfers (SETs) and multiple-embryo transfers (METs). KS distance between SET and MET of (i) test set and (ii) train set embryos was 0.17 and 0.11, respectively. KS: Kolmogorov-Smirnov. |

| 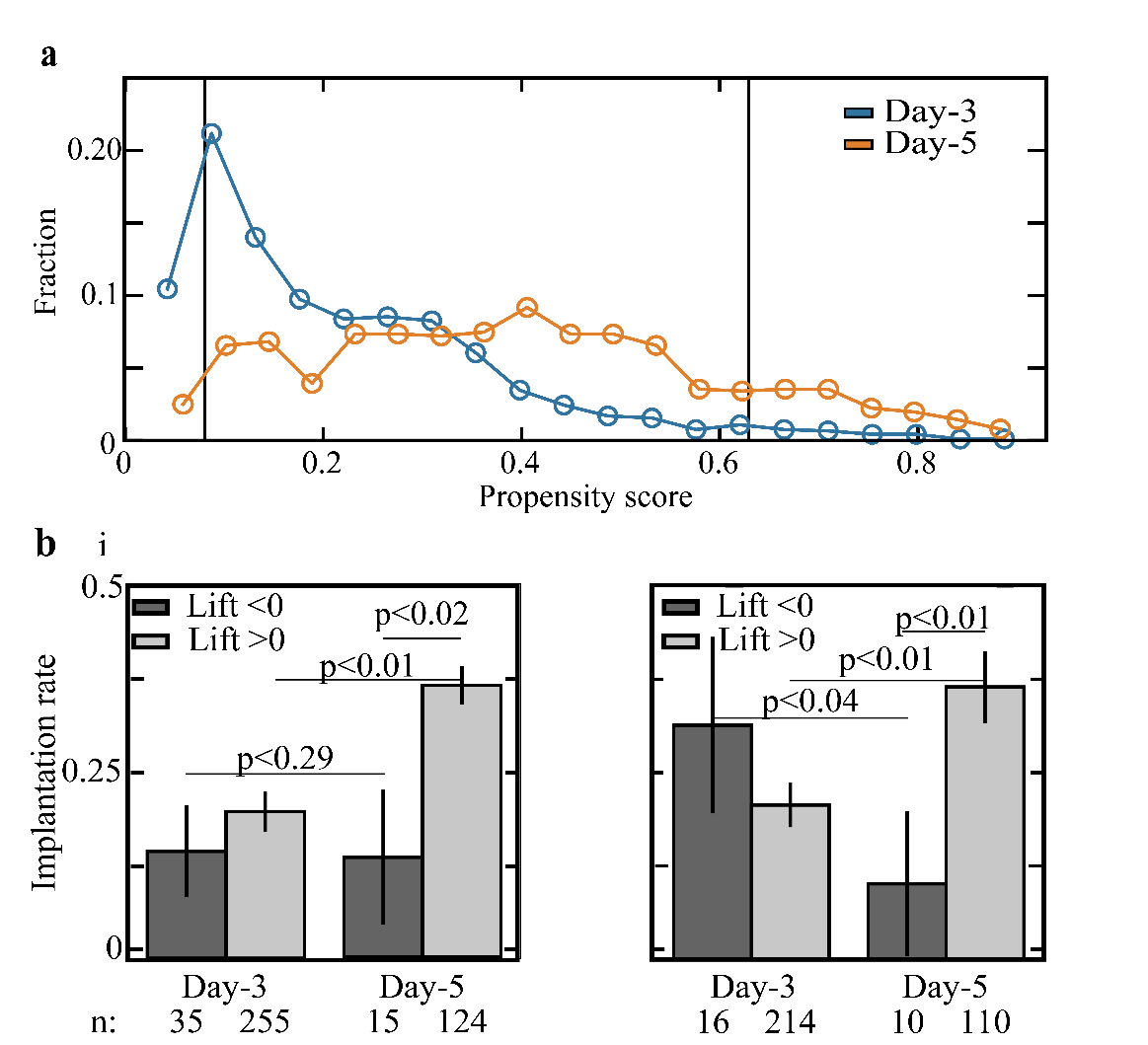 |
| --- |
| **Figure S4: The heterogeneous day-of-transfer treatment effect is independent of the site.** **A,** Day-3 and Day-5 propensity score distributions were evaluated by fitting a logistic regression prediction model of the day-of-transfer that included the clinical site dummy variable. Lower and upper bounds for excluding non-overlapping embryos are set by the 2.5 percentile of the Day-5 propensity distribution and the 97.5 percentile Day-3 propensity distribution, respectively. **B,** The average implantation rates of negative and positive *Transfer Lift* embryos that were transferred on Day-3 and on Day-5 are compared (i) for all test-set embryos, and (ii) across high-quality 8C+ embryos (at 66 hours). Error bars represent STD. |

| 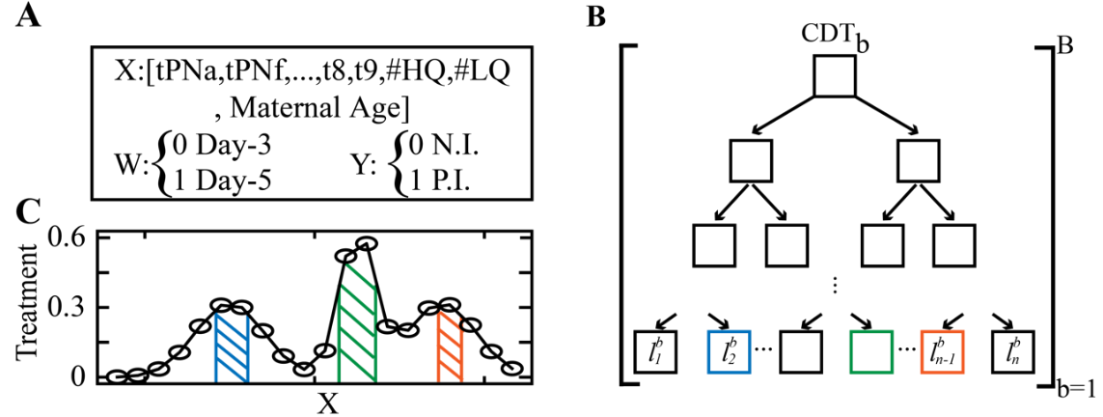 |
| --- |
| **Figure S5 \| Illustration of the causal forest model:** **A,** The counterfactual inference problem is set by the embryo feature vector $X$, the treatment indicator $W$, and the treatment response $Y$. **B,** Schematic illustration of a single causal decision tree (CDT), among a total of $B$ CDTs, illustrates the hierarchical classification of the embryos into discrete leaves. **C,** Heuristic representation depicts the variation in the Treatment $W$ between clusters of embryos (leaves) of similar feature profiles $X$. |
